# Safety and efficacy of tirofiban after early neurological deterioration in patients with branch atheromatous disease receiving alteplase

**DOI:** 10.3389/fstro.2022.968510

**Published:** 2022-10-13

**Authors:** Xuemin Zhong, Meng Zhao, Ronghua Xu, Jian Wang, Jiaxiu Du

**Affiliations:** ^1^Second People's Hospital of Chengdu, Sichuan, China; ^2^People's Hospital of Zhengzhou, Henan, China

**Keywords:** branch atheromatous disease, early neurological deterioration, GP-IIb/IIIa inhibitor, stroke, tirofiban

## Abstract

**Objectives:**

No standard treatment exists for branch atheromatous disease (BAD), and patients' conditions often worsen after thrombolytic therapy. We evaluated the safety and effectiveness of tirofiban after early neurological deterioration (END) development in patients receiving intravenous alteplase.

**Materials and methods:**

Bleeding incidence, National Institute of Health Stroke Scale (NIHSS) score, and modified Rankin scale (mRS) score were assessed for patients with BAD receiving alteplase within 4.5 h of stroke onset.

**Results:**

Among 193 patients, 119 (61.64%) did not experience exacerbation after thrombolytic treatment, 74 (38.34%) had END, 34 were treated with tirofiban after END, and 40 received standard treatment. On day 7 or at discharge, no cases of symptomatic cerebral hemorrhage were noted, and no patient died during the 90-day follow-up. Fifty-two of 74 patients (70.27%) had a good mRS score at 90 days. Among patients with END who received tirofiban, 27 (79.41%) had a good mRS score at 90 days, which was significantly better than that of the 18 cases that did not receive tirofiban after exacerbation (45%; *P* < 0.001). NIHSS scores were significantly lower 24 h, 48 h, and 7 days after tirofiban treatment in patients with exacerbation after thrombolytic therapy than in those without tirofiban treatment.

**Conclusions:**

Patients with BAD have elevated risks of END after thrombolytic therapy. Compared with conventional oral antiplatelet aggregation drugs, tirofiban rescue therapy resulted in significantly better NIHSS and mRS scores without increased symptomatic cerebral hemorrhage rates.

## Introduction

Branch atheromatous disease (BAD) is a special type of acute ischemic stroke. Caplan first proposed this term in 1989 (Caplan, 1989). It describes an internal capsule or pontine infarction caused by occlusion or stenosis of the perforator artery opening. BAD pathogenesis is mainly related to atherosclerosis, including vascular occlusion caused by carrier atherosclerotic plaque occlusion or extending to the perforator artery opening, lumen occlusion caused by an atherosclerotic plaque at the perforator artery opening or vascular occlusion caused by unstable plaque shedding (Petrone et al., 2016; Siegler et al., 2016; Shkirkova et al., 2018). Early neurological deterioration (END) is still more common in patients with BAD receiving thrombolytic therapy. Recent literature indicates that alteplase temporarily improves the clinical symptoms of patients with BAD (Rubiera et al., 2005; Saqqur et al., 2007; Uemura et al., 2014), but 57.1% of patients still have END after treatment (Deguchi et al., 2013). END is a major cause of poor outcomes in patients with stroke (Shkirkova et al., 2018).

The ARTIS trial found that early intravenous administration of aspirin 300 mg shortly after alteplase did not improve outcomes at 3 months and increased the risk of symptomatic intracranial hemorrhage (sICH) (Jeong et al., 2016). However, some studies found that tirofiban in patients with END within the first 24 h after IVT (intravenous thrombolysis) did not increase the risk of sICH, ICH, and mortality. It seems associated with neurological improvement at 3 months (Li et al., 2016; Wu et al., 2019). In this study, we evaluated the safety and efficacy of tirofiban after END in patients with BAD receiving alteplase through a retrospective analysis.

## Materials and methods

### Study design and ethical considerations

The studies involving human participants were reviewed and approved by the Ethical Review Board of the Second People's Hospital of Chengdu. Written informed consent from the patients/participants or patients/participants' legal guardian/next of kin was not required to participate in this study in accordance with the national legislation and the institutional requirements.

### Patients

This study included patients with BAD who received intravenous alteplase at Chengdu Second People's Hospital and Zhengzhou People's Hospital from January 2019 to January 2022 within 4.5 h of stroke onset. Two experienced neurologists simultaneously screened the clinical and imaging data of all patients. We excluded patients who received oral or intravenous anticoagulants at admission. Clinical data of eligible patients were extracted from the hospital database, and the following patient information was collected: (1) general information [age, sex, smoking history, drinking history, systolic blood pressure (SBP) and diastolic blood pressure (DBP)]; (2) underlying diseases (hypertension, diabetes, hyperlipidemia, and history of atrial fibrillation); (3) stroke severity assessed using National Institutes of Health Stroke Scale (NIHSS) scores at admission, 24 h, 48 h, and 7 days after treatment and at treatment conclusion; (4) modified Rankin scale (mRS) scores at admission and at 90 days after treatment to evaluate END; and (5) occurrence of bleeding events and death during treatment.

### Imaging procedures

At both centers, diffusion-weighted imaging (DWI) sequences of head computer tomography (CT) and magnetic resonance imaging (MRI) were first performed for systematic examination before thrombolytic therapy. A 3.0-T MRI scanner (Signa EchoSpeed, GE Healthcare) was used to collect fluid attenuation inversion recovery sequences, DWI, T2^*^-weighted gradient echo sequences, and intracranial 3D temporal relaxation magnetic resonance angiography. Perfusion-weighted imaging and high-resolution magnetic resonance angiography were performed when necessary. Transcranial doppler sonography (TCD) monitoring was used to assess intracranial blood flow and microemboli formation/improvement after thrombolytic therapy. The TCD foaming test was used to exclude abnormal emboli from cardiopulmonary sources. A carotid ultrasound investigation of extracranial cervical vascular stenosis and atherosclerotic plaques was performed. Transthoracic echocardiography was conducted to detect emboli of cardiac origin. Dynamic electrocardiograms were obtained for 24–72 h to detect arrhythmias, including atrial fibrillation. Head and neck computed tomography angiography was conducted to determine the degree of vascular stenosis and the possibility of arterial embolism. All patients with BAD were reexamined using head CT to exclude bleeding after END.

### Diagnostic criteria for BAD and END

The current Chinese expert consensus is that the diagnostic criteria for BAD are based on blood supply areas of perforating arteries and imaging features of the lesions. Based on this, the Japanese scholars Adachi and Takagi (Xuejiao et al., 2021) improved the diagnostic criteria for BAD in 2006.

The criteria for ischemic stroke in the lenticulostriate arteries (LSA) area were as follows: (1) diagnostic criteria for acute ischemic stroke are met; (2) DWI reveals infarct foci in corresponding blood supply area involving three levels or more; (3) blood supply areas of LSA comprise the putamen, lateral part of globus pallidus, head and body part of the caudate nucleus, forelimb of the inner capsule, the upper part of the inner capsule, and radiative corona around the ventricles. Criteria for paramedian pontine arteries (PPA) in ischemic stroke were as follows: DWI indicates an infarct connected to the ventral surface of the pons, and the lesion is located near the midline, on one side, and not beyond the midline.

Exclusion criteria were as follows: (1) imaging findings of large vessel stenosis ≥50%; (2) imaging suggestive of unstable plaques in intracranial arteries, external carotid arteries, and vertebral arteries, which could cause arterial embolization; (3) DWI revealed cortical, watershed, and multiple cerebral infarctions; or (4) cerebral infarction caused by other causes, including immune or infectious vasculitis, cardiogenic cerebral embolism, fat embolism, and platelet and coagulation dysfunction.

Most studies have defined the diagnostic criteria for END as a poor clinical outcome within 48–72 h of stroke, and NIHSS score increase of ≥2 points (≥1 point increase in motor function score) (Petrone et al., 2016; Siegler et al., 2016; Shkirkova et al., 2018).

### Criteria for safety and effectiveness

The primary outcome measure was a good mRS score (0–2) at 90 days post-stroke. The second evaluation index was stroke improvement, evaluated according to the NIHSS to assess conditions before thrombolytic therapy, at 24 and 48 h, and then 7 days after thrombolytic therapy. NIHSS score improvements were evaluated 24 h after the development of END. Scores at 48 h and 7 days were respectively subtracted from scores at the onset of END. The safety outcome was any form of bleeding or death.

### Statistical analysis

All statistical analyses were performed using SPSS version 18.0 software (IBM Corp., Armonk, NY, USA). The Kolmogorov–Smirnov test was conducted to confirm the normality of variables. Continuous and normally distributed variables are expressed as mean ± standard deviation (*x* ± *s*) and were compared using a *t*-test. Non-normally distributed variables are expressed as medians with interquartile ranges and were compared using the Kruskal–Wallis test. Categorical data are presented as frequencies and percentages, and the chi-squared test was used for comparison. Bonferroni's *post hoc* test with corrections for multiple comparisons was conducted to analyze differences between subgroups. *P* < 0.05 indicated statistical significance. Binary logistic regression models were used to evaluate the association between tirofiban infusion and the outcome variables. We included any confounding variables with a bivariate *P* < 0.1 and other selected baseline characteristics for multivariate logistic regression analysis.

## Result

### Baseline characteristics

During the study period, 193 (143 in LSA and 50 in paramedian pontine arteries (PPA) blood supply area) patients with BAD received intravenous thrombolytic therapy. Among them, 119 had no exacerbation after thrombolytic therapy, and 74 (38.34%) had exacerbation (head CT excluded bleeding) within 48 h after thrombolytic therapy. END occurred within 48 h of cerebral infarction onset in all 74 patients, and 56 of 74 (89%) patients had exacerbation within 24 h of onset. The average age of the patients was 62.03 ± 12.27 years, and the proportion of male patients was 65.80%. The median NIHSS score was 4 (1, 5), and the median mRS score was 3 (2, 4) at admission (see Table 1 for baseline information).

**Table 1:** Patient demographics and medical information.

	**No END**	**END**	***P*-value**
	***n* = 119 (61.66)**	***n* = 74** **(38.34)**	
**Baseline information**			
Age, years	62.37 ± 12.70	61.47 ± 11.60	0.623
Gender (male)	77 (64.71)	50 (67.57)	0.684
Hypertension	76 (63.87)	46 (62.16)	0.811
Diabetes mellitus	46 (38.66)	24 (32.43)	0.382
Hyperlipidemia	14 (11.76)	12 (16.22)	0.378
Current smoking	41 (34.45)	31 (41.89)	0.299
Drinking	27 (22.69)	25 (33.78)	0.091
**Clinical outcomes**			
NIHSS on admission, median (25%, 75%)	3 (2, 5)	4 (3, 6)	0.008
NIHSS after 7 days, median (25%, 75%)	2 (1, 3)	5 (3, 6.15)	<0.001
NIHSS at discharge, median (25%, 75%)	1 (0, 2)	4 (2, 6)	<0.001
mRS on admission, median (25%, 75%)	3 (2, 4)	3 (2, 4)	0.068
mRS after 7 days, median (25%, 75%)	1 (1, 2)	3(2, 4)	<0.001
mRS at discharge, median (25%, 75%)	1 (0, 2)	3 (2, 4)	<0.001
mRS at 90 days, median (25%, 75%)	0 (0, 1)	2 (1, 3)	<0.001

END, Early neurological deterioration; NIHSS, National Institute of Health Stroke Scale; mRS, modified Rankin scale.

Among 34 patients with exacerbation after thrombolytic therapy, tirofiban (tirofiban group) was administered within 12 h after excluding bleeding using head CT during the review of exacerbation. Twenty-eight patients received tirofiban after exacerbation within 24 h after thrombolysis. Tirofiban was administered intravenously at a loading dose of 0.4 μg/kg·min for 30 min (total dose not exceeding 1 mg). Intravenous delivery continued at 0.1 μg/kg·min for 48 h, followed by 4–6 h of concurrent oral antiplatelet aggregation therapy. Forty patients were not administered additional tirofiban treatment and received oral antiplatelet aggregation therapy after the exclusion of bleeding by head CT reexamination 24 h after thrombolytic exacerbation. Baseline features are presented in Table 2.

**Table 2:** Basic information and clinical outcomes of patients with END.

	**END without tirofiban**	**END with tirofiban**	**Difference**
	***n* = 40**	***n* = 34**	**(*P*-value)**
**Baseline information**			
Age, years	62.73 ± 11.67	60.00 ± 11.53	0.317
Gender (male)	23 (57.50)	27 (79.41)	0.051
Hypertension	25 (62.50)	21 (61.76)	0.948
Diabetes mellitus	12 (30.00)	12 (35.29)	0.628
Hyperlipidemia	8 (20.00)	4 (11.76)	0.338
Current smoking	17 (42.50)	14 (41.18)	0.908
Drinking	12 (30.00)	13 (38.24)	0.455
SBP on admission	149 (135, 164.75)	146.50 (135, 160)	0.394
DBP on admission	90.5 (78.25, 102)	80 (77.75, 90)	0.06
SBP after r-TPA	142.5 (129.25, 155)	146.5 (137.5, 158.75)	0.35
DBP after r-TPA	83 (75.25, 88.75)	83.5 (76, 88)	0.093
SBP on END	140 (133.25, 154)	140 (130, 156.25)	0.883
DBP on END	80 (73, 88.25)	78 (70, 80)	0.093
LSA	29	22	0.47
PPA	11	12	0.47
**Clinical outcomes**			
NIHSS			
NIHSS on admission, median (25%, 75%)	4 (3, 6)	4.5 (4, 7)	0.079
NIHSS after r-TPA, median (25%, 75%)	3 (1, 4)	4 (1, 5)	0.086
NIHSS at END, median (25%, 75%)	7 (5, 8.75)	7 (5, 9.25)	0.631
NIHSS at 24 h after END, median (25%, 75%)	7 (5, 8)	5 (3, 7.25)	0.005
NIHSS at 48 h after END, median (25%, 75%)	6.5 (5, 8)	5 (2.75, 7)	0.006
NIHSS at 7 days after END, median (25%, 75%)	6 (3.25, 7)	3.5 (2, 6)	0.005
NIHSS at discharge, median (25%, 75%)	5 (3, 6)	3 (1.75, 5)	0.007
**mRS**			
mRS on admission, median (25%, 75%)	3 (2, 4)	3 (2, 4)	0.566
mRS after r-TPA, median (25%, 75%)	2 (1, 3)	2 (1, 3)	0.942
mRS at END, median (25%, 75%)	4 (3, 4.75)	4 (3, 4)	0.223
mRS at 7 days after END, median (25%, 75%)	4 (3, 4)	3 (2, 3)	<0.001
mRS at discharge, median (25%, 75%)	3 (2, 4)	2 (1, 3)	<0.001
mRS after 90-days, median (25%, 75%)	3 (2, 3)	1 (0, 2)	<0.001
90 days, mRS (0–1)	8	19	<0.001
90 days, mRS (0–2)	18	27	<0.001
**Complications**			
Intracerebral	0	0	-
Peripheral Bleeding	3	2	0.781
Macrohematuria	3	2	0.781
Death	0	0	-

The “H value” is from the Kruskal–Wallis test.

END, early neurological deterioration; SBP, systolic blood pressure; DBP, diastolic blood pressure; LSA, lenticulostriate artery; PPA, parahippocampal place area; NIHSS, National Institute of Health Stroke Scale; r-TPA, recombinant tissue-type plasminogen activator; mRS, modified Rankin scale.

No significant differences were observed in sex, age, hypertension, diabetes, hyperlipidemia, and smoking and drinking history between the tirofiban and observation groups (*P* > 0.05). No significant differences were observed in SBP and DBP on admission, after r-TPA (recombinant tissue-type plasminogen activator), on END between the tirofiban and observation groups (*P* > 0.05). No significant differences were observed in NIHSS and mRS scores between the groups at admission, after thrombolytic therapy, and at the development of END (*P* > 0.05; Table 2).

### Comparison of safety

No patients in any group presented with sICH or severe systemic bleeding at day 7 or discharge. We identified five, three, and two cases of gastrointestinal bleeding without aggravation after thrombolytic therapy, without tirofiban after thrombolytic therapy, and with tirofiban therapy, respectively, all of which were positive in fecal occult blood tests that became negative after symptomatic treatment; however, the difference was not statistically significant (*P* > 0.05). No patients died before the 90-day follow-up.

### Comparison of NIHSS scores

**Figure 1:**
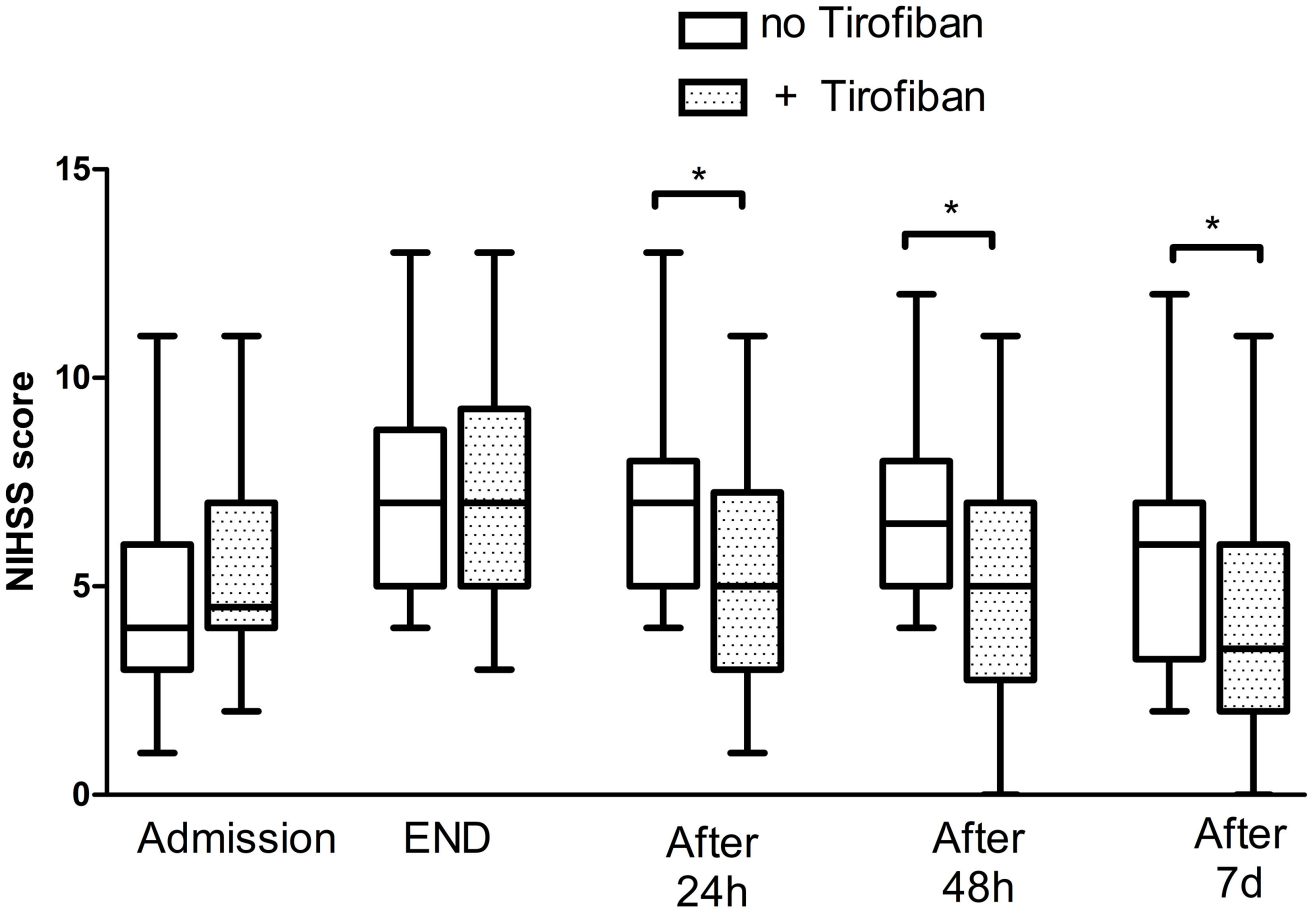
National Institute of Health Stroke Scale score of patients with END. END, early neurological deterioration; NIHSS, National Institute of Health Stroke Scale. The * symbol indicates the values of *P* < 0.05.

The median NIHSS score at 7 days was 4 (1, 5) in patients without exacerbation after thrombolytic therapy, while the median NIHSS score at 7 days was 5 (3, 6.15) in patients with exacerbation after thrombolytic therapy. Median NIHSS scores at 24 h, 48 h, and 7 days after tirofiban treatment in patients with exacerbation after thrombolytic therapy were 5 (3, 7.25), 5 (2.75, 7), and 3.5 (2, 6), respectively. This was significantly lower than that of the group aggravated after thrombolytic treatment without tirofiban, which were 7 (5, 8), 6.5 (5, 8), and 6 (3.25, 7) (*P* = 0.005, 0.006, and 0.005) at 24 h, 48 h, and 7 days, respectively. After adjusting the confounding factors (gender, NIHSS on admission, NIHSS after r-TPA by multivariable regression analysis, the patients receiving tirofiban plus rt-pa still had lower NIHSS scores at 24 h (*P* < 0.001), 48 h (*P* < 0.001), and 7 days (*P* < 0.001), respectively counterparts (Figure 1).

### Comparison of mRS scores

The median mRS score of patients with BAD was 2 (1, 3) at 7 days and 1 (0, 2) at 90 days after thrombolytic therapy. The median mRS score was 1 (1, 2) at 7 days after thrombolytic therapy and 0 (0, 1) at 90 days without recombination therapy. The median mRS score was 3 (2, 4) at 7 days and 2 (1, 3) at 90 days after thrombolytic plus recombinant therapy. The median mRS score at 90 days was 3 in the non-tirofiban group and 1 in the tirofiban group, with a statistically significant difference between groups (*P* < 0.001).

**Figure 2:**
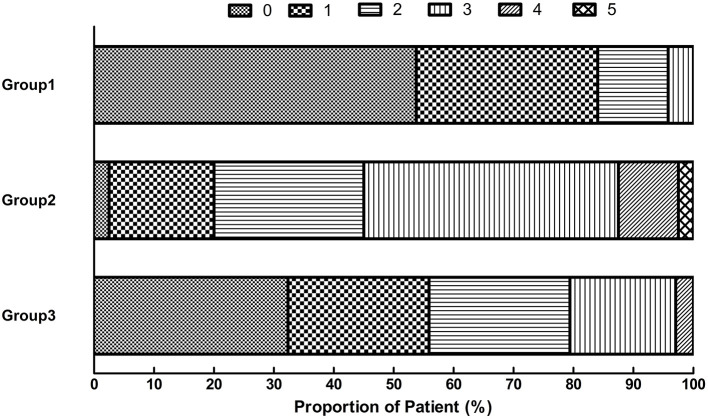
Distribution of scores on the modified Rankin scale at 90 days. Group 1: Patients without END. Group 2: END without tirofiban. Group 3: END with tirofiban. END, early neurological deterioration.

Among the 119 patients with BAD without exacerbation after thrombolytic therapy, 114 (95.8%) had a good mRS score (mRS ≤2) at 90 days. Among the 74 patients with BAD exacerbation after thrombolytic therapy, 52 (70.27%) had a good mRS score at 90 days. Among the 34 patients with exacerbation after thrombolytic therapy who received tirofiban, 27 (79.41%) had a good mRS score at 90 days, which was significantly better than the scores of 18 patients (45%) not administered tirofiban after exacerbation (Figure 2). After adjusting the confounding factors (gender, NIHSS on admission, NIHSS after r-TPA) by multivariable regression analysis, the patients receiving tirofiban plus rt-pa still had better functional outcomes (*P* < 0.001) than their counterparts.

## Discussion

In this observational study, more than one-third (38.34%) of patients with BAD developed END even after thrombolytic therapy was administered within the time window. Early tirofiban use in patients with BAD after END after IVT within 24 h did not increase the risk of sICH, ICH, severe systemic bleeding, and mortality compared with patients who did not receive tirofiban treatment. Contrarily, tirofiban rescue therapy resulted in significantly lower neurological deficits and better long-term prognosis in patients with BAD after END when compared with those without tirofiban.

Our study found a high rate of exacerbations after thrombolysis, consistent with our findings. One study reported that r-TPA did not prevent the progression of BAD during symptom fluctuations (Zhou et al., 2018). Another study suggested that r-TPA temporarily improved the clinical symptoms of patients with BAD, but 57.1% of them experienced a relapse of symptoms after treatment (Deguchi et al., 2013). END occurrence after recanalization is closely associated with cerebral edema and symptomatic bleeding (Jauch et al., 2013). However, in more than half of patients, the mechanisms underlying the deterioration of initial neurological deficits remain unclear. This type of END is referred to as unexplained END (UNEND) (Seners et al., 2014). UNEND extends from ischemic tissue in the core or penumbra region of initial neuronal necrosis to surrounding tissue without neurological defects, inducing secondary hemodynamics and dysfunctional brain tissue metabolism. This leads to the gradual aggravation of symptoms, which is currently the most widely accepted mechanism of UNEND (Tisserand et al., 2014). We assume that patients receiving thrombolysis still develop END, which might be associated with exposure to vascular endothelium after thrombolysis. However, the effect of IVT on the END of BAD patients needs to be further confirmed.

Early use of antiplatelet drugs within 24 h after IVT has always been a confusing clinical problem (The National Institute of Neurological Disorders Stroke R-TPA Stroke Study Group, 1995; Hacke et al., 2008). However, some studies found that tirofiban in patients with END within the first 24 h after IVT did not increase the risk of bleeding. It seems associated with good function at 3 months (Li et al., 2016; Wu et al., 2019). A retrospective analysis of patients with ischemic stroke in Korea also found no increased risk of hemorrhage with early initiation of antiplatelet or anticoagulant therapy (<24 h) after intravenous alteplase or endovascular treatment compared with initiation at >24 h (Liu et al., 2022). Tirofiban inhibits platelet aggregation within 5 min after intravenous injection and reaches the peak time (Dornbos et al., 2018). Steady plasma concentrations are achieved within 30 min to 1 h (Liu et al., 2019). In patients with BAD arteriosclerosis, the lumen is narrowed, and thrombi form readily after platelets adhere to the lumen, resulting in disease progression. Based on the mechanisms of action of tirofiban and thrombolytic drugs, tirofiban, in combination with thrombolytic therapy, may exert superior effects on thrombotic stroke. In recent years, growing evidence supports tirofiban as a treatment option for thromboembolic diseases (Junghans et al., 2001; Lin et al., 2017). However, clinical trials are warranted for further validation.

Our study demonstrated that tirofiban administration within 24 h after thrombolytic exacerbation in patients with BAD did not increase the risk of bleeding. Our results are consistent with a retrospective study of 278 patients, which reported that tirofiban administered early in END (within 24 h) significantly improved outcomes at 3 months without increasing the risk of bleeding. In their study, 2/121(1.7%) patients had sICH, and 7/121 (5.8%) had any ICH (Wu et al., 2019). However, no ICH was observed in our study. Unlike the study above, which enrolled patients with END after IVT, we targeted patients with END of BAD after IVT, which is strongly associated with poor treatment outcomes, and smaller lesions. Also, there was a small sample size study with 41 patients who received alteplase followed by intravenous tirofiban infusion for at least 24 h, which found that intravenous tirofiban immediately after alteplase seems to be safe and potentially more effective when compared with alteplase alone for selected patients with stroke, which refers to patients with small vessel lesions (Li et al., 2016). Our study suggests that rescue therapy with tirofiban is safe for patients with BAD exacerbation after r-TPA.

We observed that NIHSS scores of patients with exacerbation after thrombolytic therapy were significantly improved by tirofiban rescue treatment at 24 and 48 h after tirofiban administration compared with those without tirofiban treatment. Moreover, mRS scores of patients with good prognoses at 90 days were significantly better than those without tirofiban treatment. Our study's findings align with other glycoproteins IIb/IIIa antagonist studies (Junghans et al., 2001; Lin et al., 2017). Tirofiban inhibits GP-IIb / IIIa through selective competition and acts on the final link of platelet aggregation, which can rapidly direct platelet aggregation inhibition (Liu et al., 2019). Another small sample study suggested that early bridging of tirofiban after thrombolytic therapy in patients with BAD significantly improved patient outcomes without increasing the risk of bleeding (Philipps et al., 2009).

This study had several limitations. The overall sample size was small. As a retrospective study, data reliability could be lower than that of randomized controlled trials. Furthermore, we did not use neuroimaging to confirm the occurrence of reocclusion or recanalization of the artery in different conditions. We used clinical evaluation as an alternative measure, which could have errors. Moreover, the control group was administered oral antiplatelet aggregation drugs. Thus, the specific drug names and use of monoclonal or double antibodies were not analyzed in detail.

In conclusion, patients with BAD have an elevated risk of recurrence after thrombolytic therapy. Tirofiban rescue therapy resulted in significantly lower neurological deficits and better long-term prognosis in patients with BAD after END when compared with those without tirofiban, and no signs of increased intracranial bleeding were present.

## Data availability statement

The original contributions presented in the study are included in the article/Supplementary material, further inquiries can be directed to the corresponding authors.

## Ethics statement

The studies involving human participants were reviewed and approved by the Ethical Review Board of the Second People's Hospital of Chengdu. Written informed consent from the patients/participants or patients/participants' legal guardian/next of kin was not required to participate in this study in accordance with the national legislation and the institutional requirements.

## Author contributions

XZ and MZ were responsible for the concept and design of the study, data collection, and the first draft of the paper and final manuscript. RX, JW, and JD were responsible for the concept and design of the study. All authors read and approved the final manuscript for publication.

## Funding

This study was supported by the Project of Chengdu Science and Technology Bureau (2019-YF09-00097-SN).

## Conflict of interest

The authors declare that the research was conducted in the absence of any commercial or financial relationships that could be construed as a potential conflict of interest.

## Publisher's note

All claims expressed in this article are solely those of the authors and do not necessarily represent those of their affiliated organizations, or those of the publisher, the editors and the reviewers. Any product that may be evaluated in this article, or claim that may be made by its manufacturer, is not guaranteed or endorsed by the publisher.

## Abbreviations

BAD: Branch atheromatous disease
END: early neurological deterioration
sICH: symptomatic intracranial hemorrhage
SBP: systolic blood pressure
DBP: diastolic blood pressure
mRS: modified Rankin scale
DWI: diffusion-weighted imaging
CT: computer tomography
MRI: magnetic resonance imaging
TCD: transcranial doppler sonography
LSA: lenticulostriate arteries
NIHSS: National Institutes of Health Stroke Scale
PPA: paramedian pontine arteries
UNEND: unexplained END
IVT: intravenous thrombolysis
r-TPA: recombinant tissue-type plasminogen activator.
